# The Role of the U5 snRNP in Genetic Disorders and Cancer

**DOI:** 10.3389/fgene.2021.636620

**Published:** 2021-01-28

**Authors:** Katherine A. Wood, Megan A. Eadsforth, William G. Newman, Raymond T. O’Keefe

**Affiliations:** ^1^Division of Evolution and Genomic Sciences, Faculty of Biology, Medicine and Health, School of Biological Sciences, The University of Manchester, Manchester, United Kingdom; ^2^Manchester Centre for Genomic Medicine, Manchester Academic Health Science Centre, Manchester University NHS Foundation Trust, Manchester, United Kingdom

**Keywords:** disease, cancer, U5 snRNP, pre-mRNA splicing, retinitis pigmentosa, Burn-McKeown syndrome, mandibulofacial dysostosis Guion-Almeida type, spliceosome

## Abstract

Pre-mRNA splicing is performed by the spliceosome, a dynamic macromolecular complex consisting of five small uridine-rich ribonucleoprotein complexes (the U1, U2, U4, U5, and U6 snRNPs) and numerous auxiliary splicing factors. A plethora of human disorders are caused by genetic variants affecting the function and/or expression of splicing factors, including the core snRNP proteins. Variants in the genes encoding proteins of the U5 snRNP cause two distinct and tissue-specific human disease phenotypes – variants in *PRPF6*, *PRPF8*, and *SNRP200* are associated with retinitis pigmentosa (RP), while variants in *EFTUD2* and *TXNL4A* cause the craniofacial disorders mandibulofacial dysostosis Guion-Almeida type (MFDGA) and Burn-McKeown syndrome (BMKS), respectively. Furthermore, recurrent somatic mutations or changes in the expression levels of a number of U5 snRNP proteins (*PRPF6*, *PRPF8*, *EFTUD2*, *DDX23*, and *SNRNP40*) have been associated with human cancers. How and why variants in ubiquitously expressed spliceosome proteins required for pre-mRNA splicing in all human cells result in tissue-restricted disease phenotypes is not clear. Additionally, why variants in different, yet interacting, proteins making up the same core spliceosome snRNP result in completely distinct disease outcomes – RP, craniofacial defects or cancer – is unclear. In this review, we define the roles of different U5 snRNP proteins in RP, craniofacial disorders and cancer, including how disease-associated genetic variants affect pre-mRNA splicing and the proposed disease mechanisms. We then propose potential hypotheses for how U5 snRNP variants cause tissue specificity resulting in the restricted and distinct human disorders.

## Introduction

The vast majority of human genes contain introns which interrupt the coding exons. Genes are transcribed into precursor messenger RNA (pre-mRNA) which contain introns that must be removed in the nucleus, in the process of pre-mRNA splicing. Splicing joins together the coding exons to form a functional open reading frame which can be translated to make protein in the cytoplasm. Splicing is carried out by a large, dynamic, macromolecular complex known as the spliceosome, which is composed of five small uridine-rich nuclear RNAs (snRNAs) complexed with proteins to form small nuclear ribonucleoprotein complexes (snRNPs), as well as almost 200 auxiliary proteins (Jurica and Moore, 2003; Will and Luhrmann, 2011; Matera and Wang, 2014). Splicing is initiated as the U snRNPs and auxiliary proteins assemble onto the pre-mRNA guided by *cis*-acting sequences within the mRNA itself (namely the 5′ and 3′ splice sites at either end of the intron and the branch point sequence a short distance upstream of the 3′ splice site), allowing the removal of the intron and the joining together of the exons via two sequential transesterification reactions (Wang and Burge, 2008; Will and Luhrmann, 2011). There are two principal forms of spliceosomes in eukaryotes. The majority of introns (approximately 95.5%) are spliced by the major spliceosome, which contains the U1, U2, U4, U5, and U6 snRNPs. The minor, or U12-dependent, spliceosome is formed of the U1atac, U2atac, U4atac, U5 and U6atac snRNPs and is responsible for the splicing of approximately 800 introns (Turunen et al., 2013). The U5 snRNP is the only common complex in both the major and minor spliceosome. The vast majority (an estimated 95%) of human multi-exon genes are also alternatively spliced, whereby the same pre-mRNA transcript is spliced in multiple different ways to produce distinct mature mRNAs (Pan et al., 2008; Gerstein et al., 2014). Alternative splicing allows tissue-specific and/or functionally distinct isoforms of a protein and vastly increases the protein-coding capacity of the genome in higher eukaryotes (Yeo et al., 2004; Kelemen et al., 2013).

The eukaryotic split gene architecture demands for an intricate splicing regulatory network consisting of various RNA sequences, snRNP complexes and auxiliary splicing factors. Given this complexity, it is perhaps unsurprising that this stage of gene expression is highly susceptible to variants (both hereditary and somatic) which are implicated in human disorders; it is estimated that approximately 50% of human disease-causing variants affect pre-mRNA splicing (Ward and Cooper, 2010; Lim et al., 2011; Singh and Cooper, 2012; Fredericks et al., 2015; Scotti and Swanson, 2016). Splice-affecting variants include: *cis*-acting variants within the pre-mRNA sequences themselves; microsatellite expansion disorders and RNA gain-of-function; *trans*-acting variants in auxiliary splicing proteins leading to splicing factor dysregulation and/or mis-expression; and variants affecting core proteins of the spliceosome.

Disorders caused by variants which affect core spliceosome constituents (factors making up the U snRNPs) are one of the most interesting and enigmatic classes of splice-affecting variants. These disorders are relatively rare, presumably because the effects of complete loss-of-function variants are often incompatible with life, while non-coding or hypomorphic variants are more challenging to identify. Pre-mRNA splicing is a ubiquitous pre-mRNA processing step which occurs in all cells and tissues at all times, and so variants in core proteins of the spliceosome could be expected to have widespread and systemic effects on multiple tissues. However, all these U snRNP related disorders affect only one or a small number of cell or tissue types. The mechanisms underlying tissue-specific rather than pleiotropic effects arising from core spliceosome variants remain poorly understood. Current hypotheses to explain this phenomenon include a higher dependence on spliceosomal function in different tissues and/or additional (and as-yet-unknown) tissue-specific functions for certain spliceosomal proteins which are disrupted by pathogenic variants (Lehalle et al., 2015; Beauchamp et al., 2020).

A further unknown related to variants in core spliceosomal proteins is that very distinct, tissue-restricted phenotypes arise from variants within different proteins of the same spliceosomal complexes. One of the best examples of this phenomenon, and the focus of this review, is the U5 snRNP. The U5 snRNP is a large complex consisting of the U5 snRNA, a ring of seven Sm proteins and eight core protein factors (Table 1; Will and Luhrmann, 2011). During splicing by the major spliceosome, the U5 snRNP is recruited to the spliceosome as part of the U4/U6.U5 tri-snRNP, and following extensive spliceosomal remodelling the U5 snRNP associates with the pre-mRNA via interaction of the U5 snRNA loop I with the exonic sequence upstream of the 5′ splice site (Newman and Norman, 1991, 1992; Wyatt et al., 1992; Cortes et al., 1993; Sontheimer and Steitz, 1993; Newman et al., 1995; O’Keefe et al., 1996; O’Keefe and Newman, 1998; Alvi et al., 2001; McGrail and O’Keefe, 2008). Following the first transesterification step of splicing, further spliceosomal rearrangements result in the U5 snRNA loop I contacting exonic nucleotides immediately downstream of the 3′ splice site, and therefore the U5 snRNP both tethers the 5′ exon to the spliceosome after the first step of splicing and aligns the 5′ exon and 3′ exon for the second catalytic step of splicing (Newman and Norman, 1991, 1992; Wyatt et al., 1992; Cortes et al., 1993; Sontheimer and Steitz, 1993; Newman et al., 1995; O’Keefe et al., 1996; O’Keefe and Newman, 1998; Alvi et al., 2001; Turner et al., 2004; McGrail and O’Keefe, 2008; Will and Luhrmann, 2011).

**TABLE 1:** Functions of the core U5 snRNP proteins, *S. cerevisiae* homologues and their association with retinitis pigmentosa, craniofacial disorders, or cancer.

**U5 snRNP protein**	**Homologue in *S. cerevisiae***	**Function in spliceosome**	**Retinitis pigmentosa**	**Craniofacial disorder**	**Cancer**
TXNL4A	Dib1p	Prevents premature spliceosome activation?	No	Yes	No
SNRNP40	None	Protein-protein interaction, important for assembly or stability of U4/U6.U5 tri-snRNP? Function not well-characterised	No	No	Yes
CD2BP2/U5-52K	Snu40p	Interacts with PRPF6 and TXNL4A, not part of the tri-snRNP	No	No	No
DDX23/PRPF28	Prp28p	DEAD-box helicase, required for spliceosome B formation although function not well-characterised	No	No	Yes
PRPF6	Prp6	Assembly of tri-snRNP, molecular bridge between U5 snRNP and U4/U6 snRNP	Yes	No	Yes
PRPF8	Prp8p	Assembly of U5 snRNP, regulation of SNRNP200, forms catalytic centre of spliceosome	Yes	No	Yes
SNRNP200	Brr2p	Unwinding of U4/U6 snRNA duplex, activation of spliceosome	Yes	No	No
EFTUD2	Snu114p	Regulation of SNRNP200, regulation of spliceosome dissociation	No	Yes	Yes

Variants in U5 snRNP proteins have been associated with human disorders, and these disorders generally fall into two categories – retinal disorders and craniofacial disorders – with no known overlap as yet (Table 1; Lehalle et al., 2015; Ru°žičková and Staněk, 2017; Beauchamp et al., 2020). Why and how these tissue-specific and very distinct manifestations arise from variants in different, but interacting, core proteins of the U5 snRNP complex remains poorly understood. It is worth noting that variants in the spliceosome-associated factor CWC27 have recently been identified in individuals presenting with a retinal phenotype, craniofacial defects and developmental delay, indicating that the overlap of the distinct phenotypes is possible (Xu et al., 2017). However, no individuals with both retinal and craniofacial phenotypes resulting from pathogenic variants in U5 snRNP proteins have been observed so far. Additionally, links between somatic mutations in and/or altered expression of several of the U5 snRNP proteins (including those associated with craniofacial or retinal phenotypes) and human cancers have been established (Table 1). These findings indicate that human cells are highly susceptible to altered expression and/or function of certain core spliceosome proteins. The direction and magnitude of the alteration may determine the cellular dysregulation and the resulting phenotypic presentation.

## The U5 snRNP and Retinitis Pigmentosa (RP)

Retinitis pigmentosa (RP) is one of the leading causes of hereditary blindness, with an estimated prevalence of 1:4000 (Hamel, 2006). RP initially presents as night blindness (often starting in adolescence), followed by loss of peripheral vision and eventually total blindness. RP is characterised by progressive dysfunction and loss of photoreceptor rod and cone cells. It is thought that the genetic background of individuals and environmental factors play an important role in the highly variable age of onset, severity, presence of secondary symptoms and rate of disease progression in RP patients (Hartong et al., 2006; Sorrentino et al., 2016; Ru°žičková and Staněk, 2017). RP may be described as non-syndromic, where there are no other clinical features, or syndromic when RP presents with other clinical phenotypes, such as Usher syndrome where patients suffer from RP with partial or complete deafness (Daiger et al., 2013). RP may also be secondary to other systemic disorders. RP is highly heterogenous, including genetic, allelic, phenotypic and clinical heterogeneity (Daiger et al., 2013). Autosomal dominant (adRP) (30–40% of all cases), autosomal recessive (arRP) (approximately 50% cases) and X-linked (xlRP) (approximately 10%) modes of inheritance are all associated with RP (Hartong et al., 2006; Ru°žičková and Staněk, 2017). RP is very rarely inherited as a non-Mendelian phenotype (Hartong et al., 2006).

Considering both syndromic and non-syndromic forms of RP, over 100 genes have been associated with RP^1^ (Daiger et al., 2013; Diakatou et al., 2019; González-del Pozo et al., 2020; Sun et al., 2020). Many of these RP genes are expressed specifically in the retina and are involved in photoreceptor function; however, other genes associated with RP are expressed more widely in many or all human tissues (Hartong et al., 2006). Indeed, variants in at least eight spliceosome genes (six snRNP and two non-snRNP genes) are associated with RP (Table 2; Ru°žičková and Staněk, 2017). Interestingly, all the RP variants in the U snRNPs are found in the U4/U6.U5 tri-snRNP or are non-snRNP splicing factors; there have been no RP variants reported in U1 or U2 snRNP complexes to date (Boon et al., 2007). Three of these RP-associated snRNP genes – *PRPF6*, *PRPF8* and *SNRNP200* – are members of the U5 snRNP complex (Table 2). While the RP-linked variants in these three U5 snRNP genes have well-defined effects on spliceosome assembly and/or function, and result in inefficient splicing *in vitro* and *in vivo*, how and why the observed defects in splicing translate to specific retinal degeneration and an RP phenotype is not well understood. It is hypothesised that distinct groups of pre-mRNAs which have important functions in the retina are mis-spliced in spliceosome-associated RP; however, these retina-specific mis-spliced transcripts are not currently well-characterised (Ru°žičková and Staněk, 2017).

**TABLE 2:** Spliceosome protein genes associated with retinitis pigmentosa, including *S. cerevisiae* homologues, spliceosome complex, inheritance pattern, whether disease variants affect evolutionarily conserved amino acids and references.

**Spliceosome factor gene**	***S. cerevisiae* homologue**	**Spliceosome complex**	**Type of retinitis pigmentosa**	**Disease variants affect conserved amino acids**	**Selected references**
*PRPF3*	*PRP3*	U4/U6 snRNP and U4/U6.U5 tri-snRNP	Autosomal dominant	Yes	Chakarova, 2002; Martinez-Gimeno et al., 2003; Gamundi et al., 2008; Zhong et al., 2016
*PRPF4*	*PRP4*	U4/U6 and U4/U6.U5 tri-snRNP	Autosomal dominant	Yes	Benaglio et al., 2014; Chen et al., 2014; Linder et al., 2014
*PRPF6*	*PRP6*	U5 snRNP	Autosomal dominant	Yes	Tanackovic et al., 2011a; Huang et al., 2015
*PRPF8*	*PRP8*	U5 snRNP	Autosomal dominant	Yes	McKie et al., 2001; Erkenez et al., 2002; Martinez-Gimeno et al., 2003; Ziviello et al., 2005; Towns et al., 2010; Maubaret et al., 2011
*PRPF16/DHX38*	*PRP16*	Non-snRNP	Autosomal recessive	Yes	Ajmal et al., 2014
*PRPF31*	*PRP31*	U4 snRNP	Autosomal dominant	Yes	Vithana et al., 2001; Xia et al., 2004; Sullivan et al., 2006; Waseem et al., 2007; Frio et al., 2008; Jin et al., 2008; Xiao et al., 2017
*RP9/PAP1*	No homologue	Non-snRNP	Autosomal dominant	NA	Keen et al., 2002
*SNRNP200*	*BRR2*	U5 snRNP	Autosomal dominant	Yes	Zhao et al., 2009; Li et al., 2010; Benaglio et al., 2011; Liu et al., 2012; Pan et al., 2014

It has been suggested that different cell types have differing rates of transcription and translation at different stages of development, meaning that certain tissues have higher dependencies on spliceosomal function. The human retina expresses very high levels of certain housekeeping genes, as well as major and minor spliceosomal snRNAs, compared to other tissues (Cao et al., 2011; Ru°žičková and Staněk, 2017). Therefore, variants in the spliceosomal genes in RP may cause global splicing dysregulation that manifests in the retina because of its enhanced splicing activity and increased burden on the spliceosome (Tanackovic et al., 2011b). However, this hypothesis cannot be the sole explanation for the phenotypic restriction of RP; if this is true, retinal degeneration would be expected to be a phenotypic characteristic of all disorders arising from variants in core spliceosomal factors, which is not the case (Lehalle et al., 2015). Another, non-mutually exclusive, possibility is that additional (and as-yet-unknown) tissue-specific functions for certain spliceosomal proteins are also disrupted by the disorder-associated variants. However, there is currently little evidence to support this hypothesis as a causative mechanisms in RP.

### PRPF6

**FIGURE 1:**
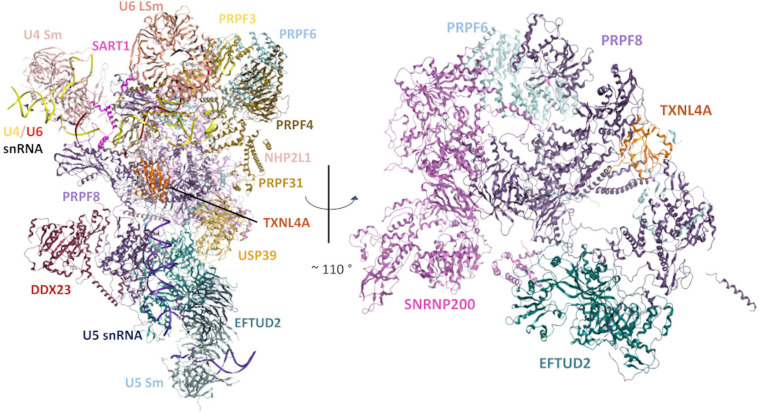
Structural model of the human tri-snRNP in two different orientations. **Left:** An overview of the human tri-snRNP, including all RNA/protein components. **Right:** Rotated view of the model, exclusively showing the structural relationships between the disorder-associated U5 snRNP proteins discussed in this review. Other tri-snRNP RNA/protein components have been omitted for clarity. Model built using a previously reported 2.9Å cryo-electron microscopy (cryo-EM) structure of the human tri-snRNP (PDB: 6QW6) (Charenton et al., 2019).

PRPF6 is a 941 amino acid, 102 kDa protein which acts as a molecular bridge between the U5 snRNP and the U4/U6 di-snRNP and is essential for the assembly of the tri-snRNP, confirmed by recent atomic structures of the human tri-snRNP (Figure 1; Makarov et al., 2000; Liu, 2006; Agafonov et al., 2016; Ru°žičková and Staněk, 2017). The first autosomal dominant RP variant reported in *PRPF6* was a heterozygous c.2185C > T (p.Arg729Trp) variant in exon 16 (Table 2; Tanackovic et al., 2011a). Arginine 729 is a highly conserved residue which lies within one of the HAT repeat domains at the C-terminal end of PRPF6. Mutant PRPF6 harbouring the p.Arg729Trp variant accumulates in Cajal bodies in patient lymphoblasts. Cajal bodies are nuclear structures involved in snRNP maturation, tri-snRNP regeneration and the site of defective snRNP accumulation (Tanackovic et al., 2011a; Staněk, 2017). Accumulation of mutant PRPF6 in Cajal bodies indicates impairment of tri-snRNP assembly in patients with the PRPF6 p.Arg729Trp variant. Indeed, the HAT domain containing Arg729 is in a region of PRPF6 known to interact with the U4/U6 di-snRNP and therefore likely affects PRPF6 interactions with this di-snRNP (Tanackovic et al., 2011a). Additionally, human cell lines with the p.Arg729Trp variant displayed inefficient splicing of a number of introns whose decreased splicing is associated with *PRPF*-linked RP in cell lines from various RP patients with *PRPF3*, *PRPF31*, and *PRPF8* variants (Tanackovic et al., 2011a,b). Therefore, the *PRPF6* p.Arg729Trp variant affects both spliceosomal composition and function.

A further two novel *PRPF6* heterozygous missense variants were identified in a cohort of Chinese patients with RP by next-generation sequencing. These variants, c.514C > T, p.Arg172Trp and c.551A > G, p.Asp184Gly, co-segregated with the disease, were absent in population variation databases and were predicted as damaging, but no further characterisation of the variants was performed (Huang et al., 2015). Furthermore, another missense variant, c.1430A > G, p.Asn477Ser, was identified within *PRPF6* co-segregating with the neurodegenerative Kufs disease. The patients with this *PRPF6* p.Asn477Ser variant were reported with visual impairment (Velinov et al., 2012). However, further work is required to confirm whether this *PRPF6* p.Asn477Ser variant has a role in either Kufs disease or the visual impairment (Ru°žičková and Staněk, 2017).

### PRPF8

**FIGURE 2:**
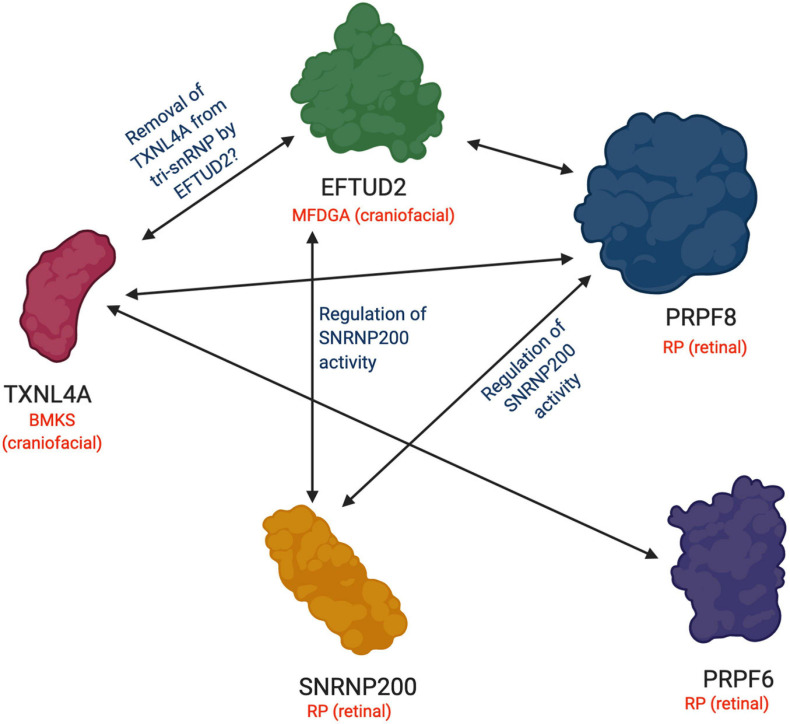
Functional and physical interactions between U5 snRNP proteins involved in retinal and craniofacial genetic disorders. A summary of the interactions between RP-associated U5 snRNP proteins and craniofacial disorder-associated U5 snRNP proteins discussed in this review. A double-headed arrow indicates an interaction between the two proteins. Blue labels describe the known function or hypothesized function of the interaction between the proteins. Genetic disorders associated with each protein are indicated in red along with the specific tissue type affected in each case. BMKS, Burn-McKeown syndrome; MFDGA; mandibulofacial dysostosis Guion-Almeida type; RP, retinitis pigmentosa. Figure created with BioRender.com.

The 220 kDa PRPF8 is the largest known protein of the spliceosome. PRPF8 is a highly conserved protein which forms the catalytic centre of the spliceosome and interacts with the U5 snRNA and the 5′ and 3′ splice sites (Galej et al., 2014; Yan et al., 2015; Bertram et al., 2017; Ru°žičková and Staněk, 2017). PRPF8 interacts with numerous tri-snRNP components including the U5 proteins, EFTUD2 (Snu114p in *Saccharomyces cerevisiae*) and SNRNP200 (Brr2p in *S. cerevisiae*), which have essential roles in the splicing cycle (Figures 1, 2). EFTUD2 and PRPF8 regulate the activity of SNRNP200, which unwinds the U4/U6 snRNA duplex to activate the spliceosome (Kuhn et al., 1999; Small et al., 2006; Frazer et al., 2008; Maeder et al., 2009; Mozaffari-Jovin et al., 2012, 2013, 2014; Nancollis et al., 2013; Nguyen et al., 2013).

At least 19 variants in *PRPF8* have been associated with autosomal dominant RP (Table 2; Malinová et al., 2017; Ru°žičková and Staněk, 2017). All the known variants cluster in the C-terminal Jab1/MPN domain of the PRPF8 protein which interacts with SNRNP200 (McKie et al., 2001; Kondo et al., 2003; Martinez-Gimeno et al., 2003; Ziviello et al., 2005; Towns et al., 2010; Malinová et al., 2017). The majority of the variants fall in exon 42, although a few *PRPF8* variants (including p.Ser2118Phe) lie within exon 38. In yeast, RP variants in *PRP8* (yeast homologue of *PRPF8*) lead to growth defects, although the growth defects were not completely consistent and may be related to the genetic background of the yeast strain (Boon et al., 2007; Maeder et al., 2009; Mozaffari-Jovin et al., 2013; Ru°žičková and Staněk, 2017). Inhibition of U5 snRNP assembly and disruption of the transition between the first and second steps of splicing were also observed in yeast models, with yeast containing Prp8p RP mutations interacting less efficiently with Snu114p and Brr2p and disrupting the regulation of Brr2p helicase activity, leading to defects in pre-mRNA splicing (Boon et al., 2007; Pena et al., 2007; Maeder et al., 2009; Mozaffari-Jovin et al., 2013; Ledoux and Guthrie, 2016; Mayerle and Guthrie, 2016). Reduced assembly of the spliceosome, inefficient splicing and differential alternative splicing was also observed in human cells derived from RP patients with *PRPF8* variants (Tanackovic et al., 2011b). *PRPF8* RP variants introduced into HeLa cells accumulated in the cytoplasm as well as displaying the normal PRPF8 nuclear localisation, while the majority of PRPF8 mutant proteins degraded more rapidly than the wildtype PRPF8 (Malinová et al., 2017). Furthermore, the majority of *PRPF8* RP variants specifically affected spliceosome assembly via inhibition of tri-snRNP formation, reducing the number of fully assembled functional spliceosomes, and consequently splicing defects were observed (Malinová et al., 2017). However, two *PRPF8* variants examined (p.Tyr2334Asn and p.Phe2314Leu) did not affect snRNP biogenesis but did affect splicing *in vivo* (Malinová et al., 2017). These p.Tyr2334Asn and p.Phe2314Leu variants inhibited SNRNP200 helicase activity and weakened association with SNRNP200, respectively, and the resulting SNRNP200 mis-regulation likely accounts for the splicing defects observed (Malinová et al., 2017).

### SNRNP200

SNRNP200 (Brr2p in yeast) is a 200 kDa protein which interacts with and is regulated by PRPF8 and EFTUD2 at the heart of the U5 snRNP (Figures 1, 2; Van Nues and Beggs, 2001; Liu, 2006; Frazer et al., 2008; Häcker et al., 2008; Mozaffari-Jovin et al., 2012; Nguyen et al., 2013). A recent 7Å cryo-electron microscopy (cryo-EM) structure of the human tri-snRNP revealed that SNRNP200 interacts with the PRPF8 Jab1 domain, the region of PRPF8 containing the majority of PRPF8 RP-linked variants (Figure 1; Agafonov et al., 2016). SNRNP200 is one of eight ATP-dependent DExD/H box RNA helicases in the spliceosome (Cordin and Beggs, 2013). SNRNP200 has an N-terminal domain of unknown function and two helicase modules, an active N-terminal helicase module which unwinds the U4/U6 snRNA duplex during spliceosome activation, and a C-terminal helicase module which acts as an intramolecular regulator (Pena et al., 2009; Zhang et al., 2009). Recent cryo-EM structures of the human spliceosomal pre-B and B complexes have indicated that as well as driving the pre-B to B complex transition during spliceosome activation, SNRNP200 undergoes a major conformational shift, resulting in a drastic change in the overall structure of the spliceosome (Zhan et al., 2018; Zhang et al., 2018).

A number of heterozygous variants in SNRNP200 have been linked to autosomal dominant RP (Table 2). The first two variants identified, p.Ser1087Leu and p.Arg1090Leu, were found in the Sec63-like domain of the N-terminal active helicase module. In yeast, both variants resulted in reduced U4/U6 snRNA unwinding (Zhao et al., 2009; Cvačková et al., 2014). In human cells, neither SNRNP200 variant affected snRNP assembly (unlike the *PRPF6* and the majority of *PRPF8* RP-linked variants) but did promote the use of cryptic splice sites (Cvačková et al., 2014). The authors proposed that SNRNP200 has an important role in 5′ splice site recognition and splicing fidelity, which is compromised by the two RP variants. Subsequently, additional SNRNP200 variants were identified in the Ski2-like helicase domain of N-terminal active helicase module, which likely disrupt the RNA helicase activity of SNRNP200 (Benaglio et al., 2011; Liu et al., 2012; Bowne et al., 2013; Zhang et al., 2013; Huang et al., 2015). Indeed, in a structural model of the SNRNP200 with a RP-associated p.Gln885Glu mutation, the affected residue is predicted to be a key nucleic acid interaction site and the change in electrostatic potential caused by the mutation would affect nucleic acid contact, disrupting U4/U6 snRNA unwinding and stalling the spliceosome (Liu et al., 2012).

These SNRNP200 RP variants suggest that mis-regulation or defects in SNRNP200 are detrimental to spliceosome activity and may disrupt RNA splicing (Ru°žičková and Staněk, 2017).

## The U5 snRNP and Craniofacial Disorders

Variants in five of the core spliceosomal proteins are associated with human disorders in which patients display abnormal craniofacial development as the primary phenotype. Two of these disorders – mandibulofacial dysostosis Guion-Almeida type (MFDGA) and Burn-McKeown syndrome (BMKS) – are caused by variants in U5 snRNP proteins, EFTUD2 and TXNL4A, respectively. These two disorders share overlapping phenotypic features, although there are unique elements to the craniofacial presentations in each case (Table 3).

**TABLE 3:** Summary of the key clinical features of mandibulofacial dysostosis Guion-Almeida type (MFDGA) and Burn-McKeown syndrome (BMKS), indicating the overlapping and unique phenotypic characteristics of patients with these syndromes.

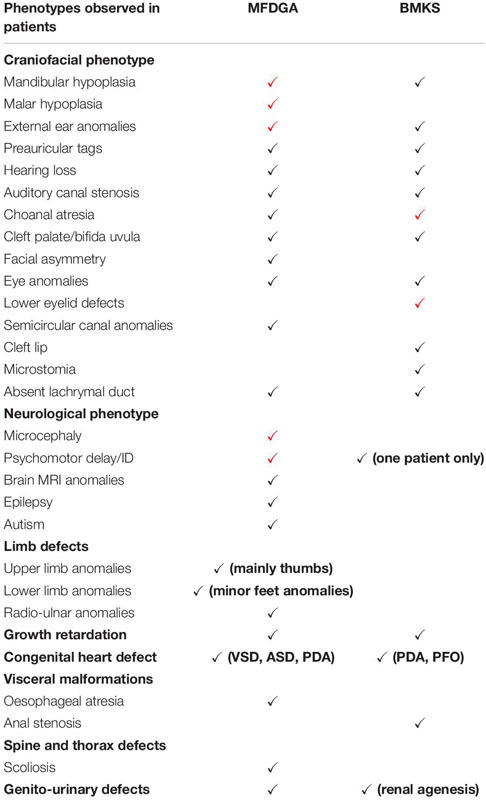

*A tick represents a feature which has been observed in at least one patient suffering from the disease. The most frequent features of each syndrome, which has been observed in more than 85% of affected individuals, are highlighted in red. ASD, atrio-septal defect; ID, intellectual disability; PDA, persistent ductus arteriosus; PFO, persistent foramen ovale; VSD, ventriculo-septal defect. Adapted from Lehalle et al. (2015); Beauchamp et al. (2020).*

The causative variants in *EFTUD2* and *TXNL4A* are predicted to lead to the mis-splicing of specific subsets of pre-mRNAs which play an important role in craniofacial development, resulting in the craniofacial defects observed in patients. This mis-splicing of disorder-relevant genes is similar to the proposed disease mechanism discussed for RP. How variants in these different proteins of the same spliceosome complex result in the mis-splicing of specific (and different) groups of pre-mRNAs affecting different developmental processes is not well-understood. Thus far, none of the patients identified with the craniofacial disorders resulting from U5 protein gene variants have presented with retinal problems, although the craniofacial defects identified in the patients are present at birth whereas retinal degeneration in RP tends to initiate later in life. It would be interesting to follow-up with MFDGA and BMKS patients in their later years to investigate whether clinical or sub-clinical retinal degeneration and vision loss has occurred.

### *EFTUD2* in Mandibulofacial Dysostosis Guion-Almeida Type (MFDGA)

Mandibulofacial dysostoses (MFDs) are craniofacial disorders in which malar and mandibular hypoplasia are the core phenotypic features; hearing loss, dysplastic ears and eyelids, and cleft palate are also frequently observed in patients (Wieczorek, 2013). In MFDGA, patients display typical MFD features, microcephaly, external ear malformations and intellectual disability. Hearing loss, cleft palate, choanal atresia, oesophageal atresia, congenital heart defects and radial ray defects are also (less commonly) observed (Table 3; Wieczorek, 2013).

In 2012, using exome sequencing approaches, Lines et al. (2012) identified heterozygous pathogenic variants in the *EFTUD2* gene as the cause of MFDGA in 12 unrelated individuals. This study, and subsequent reports, have revealed a variety of different *EFTUD2* variants, including missense variants (some of which are pathogenic by affecting splicing of *EFTUD2* pre-mRNA), nonsense variants, splice site variants and frameshifts, all of which are predicted to inactivate one allele and therefore reduce *EFTUD2* expression, supporting haploinsufficiency as the mechanism of disease (Gordon et al., 2012; Lines et al., 2012; Luquetti et al., 2013; Voigt et al., 2013; Lehalle et al., 2014; Sarkar et al., 2015; Smigiel et al., 2015; Huang et al., 2016; Vincent et al., 2016; Matsuo et al., 2017; Yu et al., 2018; Lacour et al., 2019; Thomas et al., 2020).

*EFTUD2* encodes a GTPase which is essential during multiple steps of the spliceosomal cycle, and is highly conserved across eukaryotes from yeast to humans (Fabrizio et al., 1997). Snu114p (yeast orthologue of EFTUD2) plays critical roles in spliceosomal remodelling and dynamics during pre-mRNA splicing (Frazer et al., 2008). Snu114p interacts genetically and physically with Brr2p and Prp8p (Brenner and Guthrie, 2005; Häcker et al., 2008; Nguyen T. H. D. et al., 2016). Similarly, in humans, yeast two-hybrid and *in vitro* binding assays have demonstrated physical interactions between EFTUD2, SNRNP200 and PRPF8 proteins (Figures 1, 2), and these interactions have been confirmed by recent cryo-EM structures of the human U4/U6.U5 tri-snRNP (Figure 1; Liu, 2006; Agafonov et al., 2016; Charenton et al., 2019). Prior to the first catalytic step of splicing, Snu114p is involved in the dissociation of the U4 and U6 snRNAs by regulating the activity of Brr2 (Bartels, 2002; Bartels et al., 2003; Liu, 2006; Small et al., 2006; Agafonov et al., 2016). After the splicing reactions are complete, Snu114p is also believed to regulate the dissociation of spliceosomal subunits (Small et al., 2006). Interestingly, while Snu114p/EFTUD2 interacts with both Brr2p/SNRNP200 and Prp8p/PRPF8, EFTUD2 is associated with a craniofacial phenotype while SNRNP200 and PRPF8 both cause RP (Figure 1). It is remarkable that variants in functionally and physically interacting proteins of the same spliceosome complex lead to two very different disease phenotypes.

Several groups have generated zebrafish models in which the *eftud2* gene is disrupted that display severe, disease-relevant phenotypes, indicating a vital, conserved role for *eftud2* in vertebrate development (Deml et al., 2015; Lei et al., 2017). In particular, Lei et al. (2017) developed a zebrafish model in which the *eftud2* gene contains a nonsense mutation leading to reduced *eftud2* expression. The authors found increased apoptosis and mitosis of neural progenitors but little effect on differentiated neurons in these mutants. RNA-Seq and functional analyses revealed that there was a transcriptome-wide splicing deficiency, with increased intron retention and exon skipping events, leading to inadequate nonsense-mediated decay (NMD) and activation of p53-dependent apoptosis in this *eftud2* mutant zebrafish. The authors proposed that these non-degraded, aberrant, transcripts imposed particular stresses on neural progenitors because *eftud2* expression is highly concentrated in the brain in zebrafish embryos after 36 hours post-fertilisation (36hpf) (although it was broadly expressed at earlier stages of development), promoting neural-specific apoptosis. Together, these findings demonstrated an important involvement of *eftud2* in neural progenitor development, which may contribute to the neurological abnormalities observed in MFDGA patients (Lei et al., 2017). Beauchamp et al. (2019) recently used *in situ* hybridisation to characterise *Eftud2* expression during mouse development and revealed that *Eftud2* is expressed throughout development (as expected for a core spliceosome factor), but with a particularly enrichment in the developing head and craniofacial regions. The authors also generated a mouse model with a loss-of-function exon 2 deletion in *Eftud2*; however, heterozygous embryos did not model MFDGA, while homozygous mutant embryos were not observed post-implantation, confirming a requirement for *Eftud2* expression for survival and viability of pre-implantation embryos (Beauchamp et al., 2019).

Recently, we generated an *EFTUD2* knockdown HEK293 cell line modelling *EFTUD2* haploinsufficiency in MFDGA (Wood et al., 2019). Reduction of *EFTUD2* expression in these cells resulted in decreased proliferation, cell cycle defects and an increased sensitivity to endoplasmic reticulum (ER) stress. Furthermore, RNA-Seq analysis revealed widespread mis-expression and mis-splicing of genes, including transcripts relevant to embryonic and craniofacial development, with the mis-spliced genes sharing common *cis*-acting sequence properties thought to allow (by an as-yet-unknown mechanism) increased sensitivity to dysregulated splicing when *EFTUD2* expression is lowered. We, and others, have proposed a mechanism in which an increased burden of mis-spliced pre-mRNAs in the ER resulting from reduced *EFTUD2* expression ultimately activates p53-dependent apoptosis (Wood et al., 2019; Beauchamp et al., 2020). Why cells of the developing craniofacial region are particularly affected remains to be determined, although the apparent dependence of neural progenitors on EFTUD2 in animal models is a likely explanation. Indeed, neural progenitors have very high turnover of pre-mRNAs and high levels of alternative splicing (similar to the human retina), indicative of a high reliance on spliceosome function compared to other tissues (Gurok, 2004; Rosignoli et al., 2010; Burow et al., 2015; Su et al., 2018; Weyn-Vanhentenryck et al., 2018). Additionally, neural crest cells (NCCs), the key cells involved in vertebrate craniofacial development and central to the aetiology of a number of other similar craniofacial disorders, are particularly sensitive to activated p53 and are twofold more likely to undergo apoptosis when exposed to stabilised p53 (Calo et al., 2018; Merkuri and Fish, 2019). NCCs also express higher levels of p53 than other cell types during development (Rinon et al., 2011; Merkuri and Fish, 2019). Taken together, these findings would corroborate a mechanism for MFDGA involving NCC-specific apoptosis during development.

**FIGURE 3:**
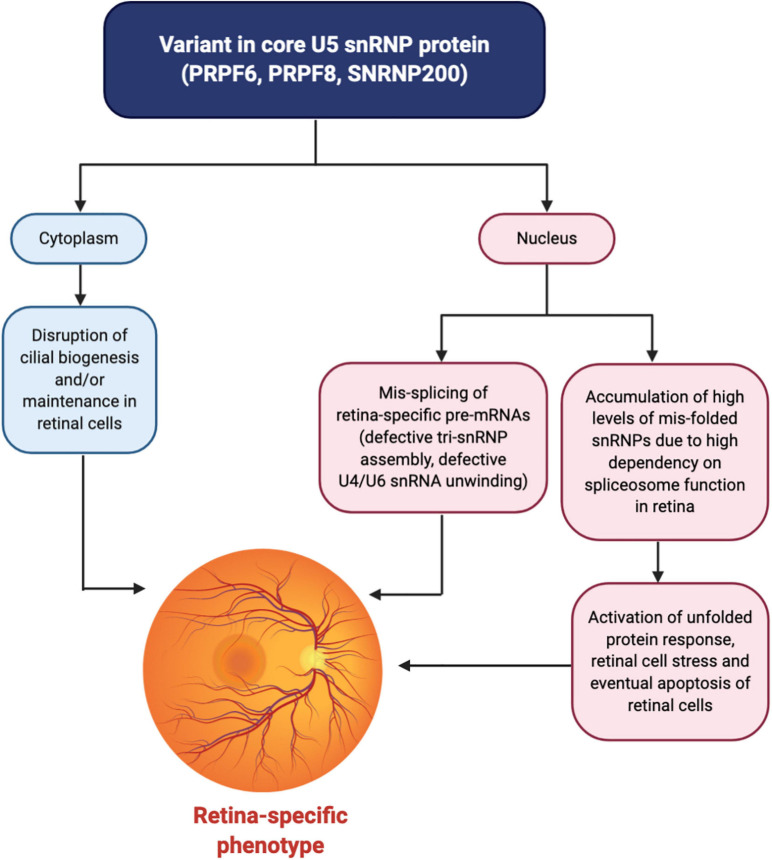
Unified mechanism for a retinal-specific phenotype caused by variants in core U5 snRNP proteins. Variants affecting the PRPF6, PRPF8, or SNRNP200 proteins result in the mis-splicing of retina-specific pre-mRNAs in the nucleus through defective tri-snRNP assembly and/or defects in U4/U6 snRNA unwinding and spliceosome activation. Why the splicing of specific pre-mRNAs is affected is currently unknown although the pre-mRNAs may share common physical features. Furthermore, an accumulation of high levels of mis-folded snRNPs in the nucleus of retinal cells, largely stemming from the increased dependency on the spliceosome in the retina due to high levels of transcription and translation compared to other tissues, activates the unfolded protein response and generates cell stress. Over time, the accumulation of cell stress, along with photo-oxidative damage to the retinal cells, triggers apoptosis of retinal cells. In the cytoplasm, additional non-spliceosomal functions of these U5 snRNP proteins in cilia biogenesis and/or maintenance may be disrupted, affecting ciliated cells of the retina. These converging mechanisms together result in retinal degeneration and an eye-specific disease phenotype. Figure created with BioRender.com.

In RP it has been suggested that constant production of mis-folded snRNP proteins over time activates the unfolded protein response and creates long-lasting stress. Together with photo-oxidative damage common to retinal cells, this stress eventually triggers apoptosis leading to retinal degeneration later in life, especially as photoreceptor cells do not regenerate so protein defects and cell stresses accumulate over time (Figure 3; Ru°žičková and Staněk, 2017). This hypothesis links the potential mechanisms of RP and MFDGA and the susceptibilities of retinal cells and NCCs to apoptosis may help to explain why these are the primary tissues affected by spliceosome protein variants.

### *TXNL4A* in Burn-McKeown Syndrome (BMKS)

Burn-McKeown syndrome is an MFD in which affected individuals display a characteristic combination of choanal atresia, craniofacial anomalies, including cleft lip and/or palate, lower eyelid coloboma, short palpebral fissures, a prominent nasal bridge, large protruding ears and sensorineural deafness (Table 3). Cardiac defects and other extra-craniofacial phenotypes may also be observed, but intellectual development is usually normal (except in one reported case thus far) (Table 3; Burns et al., 1992; Toriello and Higgins, 1999; Wieczorek et al., 2003, 2014; Lehalle et al., 2015; Goos et al., 2017; Strang-Karlsson et al., 2017; Narayanan et al., 2020). BMKS is a rare human disorder – fewer than 20 individuals with the condition have been reported, including a large consanguineous Alaskan family who were initially diagnosed with oculo-oto-facial dysplasia (Hing et al., 2006).

Wieczorek et al. (2014) identified biallelic variants in the U5 snRNP gene *TXNL4A* as causative in BMKS. Most patients have a 34 bp deletion (known as type 1Δ) in the promoter region of one allele of *TXNL4A* in combination with a loss-of-function variant (microdeletion, splice site, nonsense or frameshift variant) on the other allele (Wieczorek et al., 2014; Goos et al., 2017). Some patients are homozygous for a slightly different 34 bp deletion, the type 2Δ, in the promoter region of TXNL4A (Wieczorek et al., 2014; Narayanan et al., 2020). The type 1Δ and type 2Δ promoter deletions led to a reduction in reporter gene expression in a dual luciferase assay, with the type 2Δ causing a more severe reduction in reporter gene expression (Wieczorek et al., 2014). This more severe reduction in expression for type 2Δ might explain why a homozygous type 2Δ is sufficient to cause BMKS, while a type 1Δ must be combined with a null allele. Nonetheless, it is considered that BMKS is the product of reduced dosage of *TXNL4A* in affected individuals, with more severe genotypes such as homozygous loss-of-function variants being incompatible with life.

Interestingly, a study by Goos et al. (2017) identified two cousins with a homozygous *TXNL4A* type 2Δ with choanal atresia and other minor facial anomalies but not the full features of BMKS, in contrast to the previously described type 2Δ patients with BMKS. This finding may indicate variable or incomplete penetrance. Compensatory genetic variants in *TXNL4A* or other genes may abrogate the reduction in *TXNL4A* expression to some extent and lead to a milder phenotype.

TXNL4A is one of eight core protein members of the U5 snRNP (Liu, 2006). The *S. cerevisiae* orthologue of TXNL4A, *DIB1*, encodes a small highly conserved protein which is absolutely required for pre-mRNA splicing *in vivo*, as demonstrated by genetic depletion experiments (Reuter et al., 1999). In *S. cerevisiae*, null mutations of *DIB1* are lethal, as are deletions of the *Schizosaccharomyces pombe* orthologue *DIM1*. In haploid *S. cerevisiae* in which *DIB1* was placed under the control of the *GAL1* promoter, defective assembly of the U4/U6.U5 tri-snRNP was observed when *DIB1* expression was blocked, which was predicted to affect downstream pre-mRNA splicing (Reuter et al., 1999; Wieczorek et al., 2014). Because *DIB1* is highly evolutionarily conserved from yeast to humans, it is likely that reduced *TXNL4A* expression arising from the BMKS-associated variants in affected patients also leads to defective tri-snRNP assembly. These defects in spliceosome assembly could, in turn, lead to the altered splicing of a subset of pre-mRNAs, the downstream consequence of which is the clinical manifestation of BMKS.

Recent studies have suggested that Dib1p has an important role in preventing premature spliceosome activation, and the departure of Dib1p and other proteins from the spliceosome defines the transition from the B to B^*act*^ complex during the splicing cycle (Schreib et al., 2018). It has been proposed that Dib1p acts as a “placeholder” in the B complex, preventing formation of certain RNA-RNA interactions, the recruitment of other proteins and/or required movements to form the B^*act*^ complex. Schreib et al. (2018) generated a range of Dib1p mutants and found that Dib1p is robust and can tolerate many mutations, even at positions believed to be critical for folding stability, possibly through the compact structure of Dib1p. Dib1p also readily exchanged in splicing extracts, indicating accessibility of the Dib1p binding site in the spliceosome, despite Dib1p being a core protein of the U5 snRNP. The authors did identify two temperature-sensitive mutants which stalled *in vitro* splicing reactions before the first catalytic step of splicing and blocked spliceosome assembly at the B complex. It was proposed that the temperature-sensitivity resulted from altered interactions between Dib1p and other spliceosomal proteins, such as Prp6p and Prp8p, and not changes in Dib1p conformation (Figures 1, 2; Schreib et al., 2018). This study has provided insight into how Dib1p functions in the activation of the spliceosome. Furthermore, a recent cryo-EM structure of the human U4/U6.U5 tri-snRNP also revealed that TXNL4A is connected to EFTUD2 via PRPF8 and the U5 snRNA loop I, and it is hypothesized that EFTUD2 may catalyse the removal of TXNL4A from the tri-snRNP (Figures 1, 2; Fabrizio et al., 2009; Agafonov et al., 2016; Wan et al., 2016; Wood et al., 2019). Cryo-EM structures of the human spliceosome pre-B and B complexes also revealed that the N-terminus of PRPF6 forms two short α-helices on the surface of TXNL4A, and it is hypothesized that PRPF6 might block TXNL4A from exiting the spliceosome prematurely during the splicing cycle (Zhan et al., 2018). These findings indicate the RP- and craniofacial disorder-linked U5 snRNP proteins form an intricate network of physical and functional interactions at the heart of the spliceosome, making the phenotypic discrepancies all the more intriguing (Figures 1, 2).

Recently, we discovered that induced pluripotent stem cells (iPSCs) generated from a BMKS patient have defective differentiation to NCCs compared to maternal and unrelated control iPSCs, in particular revealing defects in the epithelial-to-mesenchymal transition (EMT) (Wood et al., 2020). RNA-Seq analysis revealed widespread differential gene expression and differential splicing in patient NCCs, with an enrichment for genes involved in processes involved in craniofacial development and the mis-splicing of a gene early in the WNT signalling pathway required for NCC specification. The mis-spliced genes shared common sequence features, although how these sequence properties render a pre-mRNA more vulnerable to mis-splicing when *TXNL4A* expression is reduced is unclear. Interestingly, the BMKS patient NCCs did not display increased apoptosis compared to maternal and control NCCs, indicating a different mechanism to that proposed for MFDGA (Wood et al., 2019, 2020). Furthermore, the mis-expressed and mis-spliced pre-mRNAs in HEK293 *EFTUD2* knockdown cells and in BMKS patient-derived NCCs did not overlap to a great extent, although again this finding may be due, at least in part, to cell type specificity, and there were different sequence features associated with the mis-spliced pre-mRNAs in each disease model (Wood et al., 2019, 2020). It would be expected that the majority of the affected transcripts in MFDGA and BMKS would be the same, resulting in the overlapping clinical features in the two disorders. Nonetheless, the differences in affected transcripts could explain the non-identical phenotypes of MFDGA and BMKS, although for a true comparison between the disorders iPSCs should be generated from MFDGA patients and differentiated to NCCs as a disorder-relevant cell type-specific model.

Therefore, similar to the discussion for RP, the variants in the core U5 snRNP proteins linked to craniofacial disorders result in changes in pre-mRNA splicing. These defects in pre-mRNA splicing are presumed to result in the specific disease phenotype at least in part, by affecting transcripts involved in the relevant developmental processes. Additionally, global mis-splicing and cell type-specific apoptosis may also play a role in the disease mechanism, at least for MFDGA. However, distinct groups of genes are presumably mis-spliced in RP and craniofacial disorders which leads to the different phenotypic features of each disorder. Understanding why specific sequence features render certain pre-mRNAs more vulnerable to mis-splicing when different U5 snRNP proteins are mutated or have reduced expression may be the key to understanding how the distinct phenotypic differences arise. It is plausible that, as every pre-mRNA is unique, certain spliceosomal proteins have a more important role in the splicing of pre-mRNAs with particular features, making those pre-mRNAs more reliant on proper functioning or amount of that spliceosomal protein for normal splicing. However, there is still much work needed to unravel this hypothesis.

## The U5 snRNP and Cancer

While variants leading to altered function and/or expression of U5 snRNP proteins are linked to retinitis pigmentosa and craniofacial disorders, links between the U5 snRNP and human cancers have also emerged. Alterations in the splicing process has been implicated in a large number of cancers, and cancer cells exploit RNA splicing to promote tumorigenesis. Aberrant alternative splicing is now considered a hallmark of cancer as cells move through the oncogenic process. Cells gain proliferative ability, become angiogenic, invasive and antiapoptotic, achieve growth factor independence, display altered metabolism to overcome hypoxia, evade the immune system, undergo an epithelial-to-mesenchymal transition and become metastatic as they become oncogenic, all of which require a switch in pre-mRNA splicing (Hanahan and Weinberg, 2000, 2011; Oltean and Bates, 2014).

Recurrent somatic mutations in spliceosome proteins and/or dysregulated expression of RNA binding proteins involved in splicing contribute to mis-splicing of transcripts which promote cancer growth and progression (Wang and Aifantis, 2020). For example, frequent heterozygous somatic missense mutations in *SRSF2*, *SF3B1, ZRSR2* and *U2AF1* have been identified in many cancers, especially in certain subtypes of leukaemia (Wang et al., 2011; Yoshida et al., 2011; Graubert et al., 2012; Yoshida and Ogawa, 2014; Anczuków and Krainer, 2016; Wang and Aifantis, 2020). These splicing factor mutations lead to changes in RNA splicing patterns, including global dysregulation of splicing, mis-splicing of subsets of genes involved in critical cell signalling pathways involved in tumorigenesis, and the promotion of tumorigenic isoforms of specific pre-mRNAs such as *BRD9* (promotes tumours growth) and *IRAK4* (hyperactivation of NF-κB signalling) (Inoue et al., 2019; Visconte et al., 2019; Wang and Aifantis, 2020). Cancer cells can also have mis-expression of RNA binding proteins, resulting in dysfunctional splicing patterns and tumour-specific dependencies (Wang and Aifantis, 2020).

Initially, the spliceosome proteins found to be commonly mutated in human cancers were all associated with the U1 and U2 snRNPs. However, somatic mutations in *PRPF8* have now been linked to myeloid neoplasms, while altered expression levels of several other U5 snRNP proteins (*PRPF6*, *EFTUD2*, *SNRNP40*, and *DDX23*) have been associated with human cancer (Table 4). *PRPF6*, *PRPF8*, and *EFTUD2* have all been associated with genetic disorders (RP and MFDGA, respectively), and in all cases it is proposed that the causative variants result in the mis-splicing of particular pre-mRNAs relevant for the disorder phenotype. However, somatic mutations and/or changes in expression of these same spliceosome proteins in cancers are also proposed to result in the dysregulation of splicing of pre-mRNAs which promote tumorigenesis. How and why different mutations and/or different expression levels of the same gene can result in such contrasting phenotypes as retinal or craniofacial defects and cancer is an enigma. In particular, precisely how and why different subsets of genes appear to be mis-spliced in each case is unclear. It may be that different types of variants and/or variants affecting different functional domains of the same spliceosomal protein alter the interaction with different classes of pre-mRNAs, depending on the characteristics of the specific pre-mRNAs, but more evidence is required to support this hypothesis. Nonetheless, these findings indicate that human cells are exquisitely sensitive to the expression level and function of core U5 snRNP factors, and any deviation can have major consequences in terms of specific rare disease phenotypes and/or cancer. The direction or magnitude of the deviation likely plays an imperative role in governing the phenotypic outcome in patients.

**TABLE 4:** U5 snRNP proteins linked to human cancer via somatic mutation or dysregulation of expression, and their association with other disorders.

**U5 snRNP protein**	**Cancer association**	**References**	**Genetic disorder association**
PRPF6	Overexpression/amplification in colon cancer	Adler et al., 2014	RP
PRPF8	Recurrent somatic mutations in myeloid neoplasms	Kurtovic-Kozaric et al., 2015	RP
EFTUD2	Overexpression in colitis-associated cancer	Lv et al., 2019	MFDGA
SNRNP40	High inter-cell expression variability in breast cancer cells, low expression associated with metastatic outcomes	Nguyen A. et al., 2016	None
DDX23	Overexpression in gliomas	Yin et al., 2015	None

*RP, retinitis pigmentosa, MFDGA, mandibulofacial dysostosis Guion-Almeida type.*

### PRPF8

In addition to the role of *PRPF8* in RP, recurrent somatic mutations and hemizygous deletions have been identified in *PRPF8* in myeloid neoplasms including myelodysplastic syndrome (Table 4) (MDS). Kurtovic-Kozaric et al. (2015) screened a large cohort of patients with MDS and related conditions to identify a number of somatic missense and somatic nonsense mutations in *PRPF8*, as well as numerous cases containing the deletion of one copy of the *PRPF8* locus exhibiting *PRPF8* haploinsufficiency (Makishima et al., 2012; Kurtovic-Kozaric et al., 2015). The *PRPF8* missense mutations are distributed throughout the length of the gene, including the Jab1/MPN domain, and were most frequently identified in primary and secondary acute myeloid leukaemia (AML), suggesting an association with more aggressive cancer phenotypes compared to low-risk MDS. *PRPF8* mutations resulted in increased cellular proliferation, and *PRPF8* mutations and deletions correlated with the presence of ringed sideroblasts (RS) and pseudo Pelger-Huet anomaly (PHA) (Kurtovic-Kozaric et al., 2015). The authors suggested that the identified *PRPF8* mutations alter the internal dynamics of the spliceosome, and revealed that splicing patterns and splice site recognition were altered in both yeast and human cells carrying the MDS-associated *PRPF8* mutations (Kurtovic-Kozaric et al., 2015). Gene expression patterns were also altered in *PRPF8* mutated and deleted samples, with many of the differentially expressed genes and mis-spliced genes associated with mitochondrial function and haematopoietic differentiation (Kurtovic-Kozaric et al., 2015). More recent work has found that *PRPF8* missense mutations in MDS patients are generally secondary mutations, and often co-occurred with other more common cancer splicing factor mutations (*SRSF2, SF3B1, LUC7L2, U2AF1*, and *ZRSR2*) (Adema et al., 2017).

Precisely how and why these *PRPF8* mutations impact the overall function of PRPF8 protein resulting in neomorphic splicing activity and leading to the malignant phenotype of aggressive myeloid malignancies with increased RS is not yet understood (Ru°žičková and Staněk, 2017). It seems likely that the mis-expression and mis-splicing of genes involved in iron accumulation in the mitochondria and abnormal haematopoiesis has a central role in the cancer phenotype (Kurtovic-Kozaric et al., 2015). Furthermore, how different missense changes can cause such a dramatically different phenotype to other missense changes in the same protein (cancer versus retinitis pigmentosa) is not known. While both *PRPF8* mutations in MDS and *PRPF8* variants in RP alter RNA splicing and change splicing patterns, why different groups of genes (presumably with different physical properties) are specifically affected by the different missense mutations in the same protein and how these result in the very different disease presentations is not known. One hypothesis is the cancer-associated variants affect the entire length of the PRPF8 protein and so may affect different functions and/or interactions of PRPF8 than the RP-linked PRPF8 variants which are only found in the Jab1/MPN domain (Kurtovic-Kozaric et al., 2015; Ru°žičková and Staněk, 2017). However, mutations affecting the Jab1/MPN domain have been identified in some cancers as well, so this explanation cannot fully account for the different disease presentations (Kurtovic-Kozaric et al., 2015). Even within the Jab1/MPN domain, different amino acids may be involved in different functions so the exact identity of the altered residue likely has an important role in disease outcome.

### PRPF6

In addition to its role in RP, overexpression or amplification of *PRPF6* is a common oncogenic driver of proliferation in human primary and metastatic colon cancer (Table 4; Adler et al., 2014; Lokody, 2014). Knockdown of *PRPF6* expression in human cancer cell lines with increased levels of PRPF6 inhibited cell growth *in vitro*, and inducible knockdown of *PRPF6* in xenograft tumours led to tumour shrinkage only in tumour models with high *PRPF6* expression (Adler et al., 2014). Reduced *PRPF6* led to intron retention of a relatively small subset of genes, including an oncogenic long isoform of the ZAK kinase (ZAK-LF) (Adler et al., 2014). ZAK-LF levels correlated with *PRPF6* expression in colon cancer cells, and *PRPF6* was required for the alternative splicing of ZAK to produce ZAK-LF. Expression of ZAK-LF transformed immortalised murine fibroblasts and induced xenograft tumour formation in immunodeficient mice, while depletion of ZAK-LF reduced the growth of *PRPF6*-overexpressing colon cancer cells *in vitro* and in xenografts (Adler et al., 2014; Lokody, 2014).

From this study, it was suggested that overexpression of *PRPF6* has an important role in driving colon cancer, via the altered splicing of gene isoforms related to growth and proliferation (Adler et al., 2014). Why different groups of genes may be differentially spliced from *PRPF6* overexpression compared to RP missense variants in PRPF6, remains unclear. Characterising the sequence properties of the mis-spliced RNAs in each case may help to begin unravelling this difference.

### SNRNP40

The function of SNRNP40 in the U5 snRNP complex during pre-mRNA splicing is not well-understood. In 2016, Nguyen et al., identified clonal human breast cancer subpopulations with different levels of morphological and molecular diversity (which are associated with metastatic colonisation and chemotherapeutic survival), and identified genes with high inter-cell transcript expression variability (Nguyen A. et al., 2016). The authors found high variability in genes encoding splicing machinery proteins, including *SNRNP40* (Table 4). The authors engineered cells with variable *SNRNP40* expression and revealed that *SNRNP40* depletion promoted systemic metastasis, with increased levels of unspliced pre-mRNAs in cells with low *SNRNP40* expression. Clinically, low *SNRNP40* expression was found associated with metastatic outcomes. It was proposed that deregulation of splicing factors, including *SNRNP40*, may amplify alterations of gene regulatory and expression networks and might lead to molecular and phenotypic diversity associated with metastasis (Nguyen A. et al., 2016). However, the underlying mechanisms of precisely how variable expression of splicing factors, including *SNRNP40*, promote metastatic progression and the overall contribution to cancer progression is not understood.

### EFTUD2

While reduced expression of *EFTUD2* is associated with MFDGA, increased expression of *EFTUD2* has been linked to human colitis-associated cancer (CAC) (Table 4). This contrast in phenotypes indicates that human cells are very sensitive to alterations in *EFTUD2* expression levels. EFTUD2 plays a role in preventing hepatitis C virus (HCV) by upregulating expression via splicing of interferon-stimulated genes (ISGs) such as RIG-I and MDA5, suggesting EFTUD2 is a novel innate immune system regulator (Zhu et al., 2015). More recently, in mouse models of CAC, *Eftud2* was overexpressed in colonic tissues and infiltrating macrophages (Lv et al., 2019). Myeloid-specific knockout of *Eftud2* suppressed chronic intestinal inflammation and tumour development by decreasing inflammatory cytokine and tumorigenic factor production via compromised activation of NF-κB signalling. This impaired signalling activation resulted from changes in *Eftud2*-mediated alternative splicing of components of the NF-κB pathway in macrophages. The authors concluded that overexpression of *Eftud2* is involved in the pathogenesis of CAC by modulating the inflammatory response of macrophages, highlighting the link between inflammation, cancer and alternative splicing in the innate immune system. Furthermore, this work emphasises how excessive *EFTUD2* expression can also lead to distinct pathological consequences compared to reduced *EFTUD2* expression in MFDGA. Why the processing and/or expression of different groups of genes is affected when *EFTUD2* is overexpressed, resulting in such a different phenotype, compared to *EFTUD2* knockdown cells modelling MFDGA remains to be determined (Wood et al., 2019). It is possible that different levels of EFTUD2 result in the formation of different splicing complexes and/or that more or less EFTUD2 protein allows either faster or slower regulation of SNRNP200, which in turn could affect the splicing of different pre-mRNAs.

### DDX23

DDX23 is an 820 amino acid DEAD-box RNA helicase protein of the U5 snRNP which is required for the formation of the spliceosomal B complex after its phosphorylation by SRPK2 (Mathew et al., 2008). However, the exact role of DDX23 in the spliceosome is not well-understood. Yin et al. (2015) identified *DDX23* overexpression in glioma tissues, with high expression of *DDX23* correlating with poor glioma patient survival (Table 4). The authors found that knockdown of *DDX23 in vitro* and *in vivo* suppressed glioma cell proliferation and invasion. Interestingly, the DDX23 protein promoted the post-transcriptional biogenesis of microRNA mir-21 via interaction with the Drosha complex (Yin et al., 2015). miR-21 upregulation was previously known to be strongly associated with proliferation, invasion and radiation resistance of glioma cells (Kwak et al., 2011; Ha and Kim, 2014). Mutagenesis demonstrated that the helicase activity of DDX23 was essential for processing of mir-21. Furthermore, inhibiting DDX23 activity chemically with the RNA helicase inhibitor ivermectin decreased miR-21 levels and blocked invasion and cell proliferation in glioma cell lines and decreased glioma growth in mouse xenografts (Yin et al., 2015). Thus, unlike other U5 proteins associated with cancer, the key role of DDX23 upregulation in glioma progression does not appear to stem from specific alterations in pre-mRNA splicing, but rather through additional functions of the protein, in this case microRNA processing. It is possible that additional, non-spliceosomal and as-yet-unknown functions of the other U5 snRNP proteins may play a role in the pathogenesis of cancer, retinitis pigmentosa or craniofacial disorders.

Interestingly, the phosphorylation of DDX23 by SPRK2 following pausing of RNA polymerase II during transcription plays an important role in suppressing R-loops, nucleic acid structures generated during transcription that can lead to genomic instability (Skourti-Stathaki and Proudfoot, 2014; Sollier and Cimprich, 2015). The absence of either SPRK2 or DDX23 leads to an accumulation of R-loops resulting in massive genomic instability, with the role of DDX23 in suppressing R-loops not requiring a functional U5 snRNP (Achsel et al., 1998; Makarov et al., 2000; Grainger and Beggs, 2005; Agafonov et al., 2016; Sridhara et al., 2017). *DDX23* mutations and homozygous deletions have been identified in several different cancers, including adenoid cystic carcinoma (ACC), implicating *DDX23* loss as a potential source of genomic instability which may have an important role in cancer development (Sridhara et al., 2017). Again, this link between *DDX23* and cancer is not related to its function in the spliceosome and argues that extra-spliceosomal functions of certain U5 proteins could potentially play a role in their pathogenesis.

## Discussion

Here we have reviewed the association between the U5 snRNP and human disease. In particular, the tissue-specific and distinct phenotypic consequences of genetic variants in different, but interacting, proteins of the same spliceosomal complex – RP and craniofacial disorders – remains arguably the biggest enigma in this field. Furthermore, the association of certain U5 snRNP proteins with cancer, including proteins also linked to RP or craniofacial defects, introduces an additional layer of complexity as mutations in and/or altered expression levels of the same protein can have very different phenotypic outcomes.

For both RP and the craniofacial disorders MFDGA and BMKS, much evidence from disease modelling supports the mis-splicing of distinct subsets of genes which may be involved in retinal function or craniofacial development, respectively. It may be that at least some of these mis-spliced genes are predominantly or only expressed in the retina or NCCs and are vitally important in development of that tissue, meaning these tissues are the more sensitive to mutation in the U5 snRNP and most affected phenotypically. The pathways affected by the mis-splicing events may also have a greater role in the development of certain tissues than others. Retina-specific mis-spliced transcripts have not yet been identified in *PRPF6*, *PRPF8*, and *SNRNP200*-associated RP, although data from *PRPF31*-defective RP patient retinal pigment epithelium (RPE) and retinal organoids has identified retinal-specific mis-splicing events (Ru°žičková and Staněk, 2017; Buskin et al., 2018). In patient-derived NCCs modelling BMKS, defects in the WNT signalling pathway were observed, which was attributed to the mis-splicing of a key WNT pathway gene (Wood et al., 2020). WNT signalling is critical in NCC specification *in vitro* and *in vivo*, and while the WNT pathway is also involved in the development of other tissue types, it was proposed that WNT signalling is proportionally more important for NCC development than in other tissues and/or the branch of the WNT pathway particularly affected by the mis-splicing event is more active in developing NCCs than other tissue types. Both the retina and developing craniofacial tissue appear to have a greater dependence on spliceosomal function than other tissues, and are thus more likely to be affected by spliceosomal dysfunction (Gurok, 2004; Rosignoli et al., 2010; Cao et al., 2011; Tanackovic et al., 2011b; Burow et al., 2015; Su et al., 2018; Weyn-Vanhentenryck et al., 2018). However, as the retinal degeneration and craniofacial defects are completely distinct, at least for U5 snRNP variants, it argues that a simple increased requirement for the spliceosome cannot be the complete answer for the phenotypic tissue specificity. However, there are further clues. For example, the proposed mechanism for MFDGA involving accumulation of mis-spliced pre-mRNAs and mis-folded proteins triggering ER stress and apoptosis, which particularly affects the activated p53 sensitive NCCs, is an intriguing explanation for tissue-specificity (Wood et al., 2019; Beauchamp et al., 2020). However, there is no evidence of this mechanism also holding true for BMKS at present.

A further hypothesis for tissue specificity of disorders arising from core U5 snRNP variants can be derived from ribosomopathies. Disorders including Diamond-Blackfan anaemia, Shwachman-Diamond syndrome and isolated congenital asplenia are all tissue-specific disorders caused by haploinsufficiency of genes involved in ribosome synthesis or function (Draptchinskaia et al., 1999; Boocock et al., 2003; Bolze et al., 2013). It has been proposed that the expression and/or activity of various ribosome proteins is not the same in all cell types, meaning that different ribosome proteins may be more or less important in different tissue types (Xue and Barna, 2012; McCann and Baserga, 2013; Lehalle et al., 2015). The same may be true for the spliceosome, with cell type specificity of spliceosome function. For example, it is possible that *PRPF6*, *PRPF8*, and *SNRNP200* are more highly expressed than other spliceosomal proteins in the retina and play a more important role in splicing in photoreceptors, while *EFTUD2* and *TXNL4A* are relatively more important in NCCs. Profiling the expression levels of the individual U5 snRNP proteins in different tissues at different stages of development would allow an investigation of the phenomenon of tissue-specific spliceosomes. That said, as core spliceosome factors the roles of each of these proteins in the spliceosome does seem to be universally important – for example, unwinding of the U4/U6 snRNAs (by SNRNP200) would be considered to be an essential step in splicing and no more or less important for one pre-mRNA than another. However, the speed of U4/U6 snRNA unwinding may influence splicing decisions. Slow unwinding and activation of a particular spliceosome may favour one splicing pattern over another. If a particular exon is usually spliced out rapidly after it has been transcribed, slowing down unwinding and delaying spliceosome activation may allow time for another exon to be transcribed and spliced instead, similar to the effect of transcriptional speed on alternative splicing patterns (Alpert et al., 2017; Pai et al., 2017; Tellier et al., 2020). Therefore, subtle differences in ubiquitous reactions in the splicing cycle could lead to differential splicing choices. It is interesting to note that causative variants in the gene inosine monophosphate dehydrogenase 1 (*IMPDH1*), which controls the rate-limiting step in GTP production, are also linked to autosomal dominant retinitis pigmentosa (Bowne et al., 2002; Aherne et al., 2005; Mortimer and Hedstrom, 2005; Spellicy et al., 2010; Bennett et al., 2020). Since *EFTUD2* is a GTPase, a common connection could be GTP availability in the retina, although this hypothesis does not account for why variants in *EFTUD2* do not manifest in a retinal phenotype.

The evidence suggests that the subsets of pre-mRNAs which are mis-spliced in RP, MFDGA and BMKS have different conserved sequence properties which makes them more vulnerable to mis-splicing when the corresponding splicing factor genes are mutated or mis-expressed (Wickramasinghe et al., 2015; Lei et al., 2017; Wood et al., 2019, 2020). Why particular sequence properties make certain pre-mRNAs more vulnerable to alterations in a specific U5 snRNP factor are not clear, but may be the key to unravelling why only certain pre-mRNAs are affected. For MFDGA, it has been suggested that the sequence features of certain exons make them more difficult to splice (less easily recognised by the spliceosome), and reducing the expression of *EFTUD2* tips the balance and results in aberrant exon skipping (Wood et al., 2019). A similar mechanism is likely true for BMKS and RP, but again this raises the question of what links certain pre-mRNA *cis* features to alterations in specific U5 proteins.

Finally, the links between the U5 snRNP proteins and cancer highlights pleiotropic phenotypic consequences arising from different mutations in, and/or different expression levels of, the same U5 snRNP component. *PRPF8* is the only U5 snRNP protein where recurrent somatic mutations have been linked to cancer, joining the ranks of spliceosome factors such as *U2AF1* and *SF3B1* as frequently mutated in certain types of human cancers, in particular leukemias (Grosso et al., 2008; Yoshida and Ogawa, 2014; Visconte et al., 2019). Why and how different groups of pre-mRNAs are affected by different missense mutations in the same protein in RP and cancer is not at all understood. It is true that *PRPF8* has multiple roles in the splicing cycle, including assembly of the tri-snRNP and regulation of SNRNP200 activity (Grainger and Beggs, 2005). One can postulate that RP-associated variants and cancer-linked somatic mutations affect different aspects of PRPF8 function, although RP *PRPF8* variants can affect both spliceosome assembly and SNRNP200 regulation (Ru°žičková and Staněk, 2017). Similarly, while haploinsufficiency of *EFTUD2* causes MFDGA, overexpression of *EFTUD2* is linked to CAC, and it is again thought that different groups of pre-mRNAs are affected in each case (Lines et al., 2012; Lv et al., 2019). It appears that the spliceosome is exquisitely sensitive to the expression levels and function of EFTUD2 and other U5 snRNP proteins, and tipping the balance in either direction can disrupt homeostasis and have pathogenic consequences. Finally, it is interesting that the link between DDX23 and glioma progression stems from a non-spliceosomal role of the protein (Yin et al., 2015). Similarly, the links between DDX23 and genomic instability related to its role in suppressing R-loops is not connected to its function in splicing (Sridhara et al., 2017). It may be worth investigating whether any of the other U5 snRNP proteins associated to human disease or cancer have roles outside of their canonical function in the spliceosome which are disrupted by pathogenic variants and could link to the observed phenotypes. Intriguingly, there is growing evidence suggesting that spliceosome proteins have a role in cilia function (Wheway et al., 2015; Kim et al., 2016; Johnson and Malicki, 2019). The photoreceptor outer segment is a specialized primary cilium while the retinal pigment epithelium is a ciliated monolayer epithelium, and variants in a number of ciliary proteins, including *RPGR*, cause retinitis pigmentosa (Ghosh et al., 2009; Parmeggiani et al., 2011; Wheway et al., 2014). Interestingly, in retinal organoids derived from *PRPF31*-defective RP patients, there was an enrichment for differentially expressed genes related to the ciliary membrane and the primary cilium, while fibroblasts from the same *PRPF31* RP patients had significant mis-splicing of genes involved in ciliogenesis (Buskin et al., 2018). Both *PRPF6* and *PRPF8* have been identified as genes which may be important in the biogenesis and/or maintenance of the primary cilium in siRNA screens (Wheway et al., 2015). Furthermore PRPF6, PRPF8 and SNRNP200 all localize to the ciliary basal body or the centrosome, outside the nucleus, indicating additional cytoplasmic roles for these U5 snRNP proteins in cilia biology unrelated to their functions in splicing (Johnson and Malicki, 2019). Therefore, defects in the formation or maintenance of the cilia in the eye caused by the variants in the core spliceosome factors could, at least in part, link genotype to phenotype. Perhaps cilial defects in combination with the mis-splicing of retinal-specific pre-mRNAs and an overall increased burden on spliceosomal activity in the retina together result in a tissue-specific phenotype (Figure 3). Furthermore, links have been identified between components of the spliceosome, including the U5 snRNP components EFTUD2 and SNRNP200, with cohesin (Kim et al., 2019). The depletion of splicing factors including EFTUD2 and SNRNP200 in HeLa cells resulted in mitotic arrest, indicating the interaction of cohesin with these splicing factors is required for mitotic progression (Kim et al., 2019). The link between U5 snRNP components, cohesin and the cell cycle may be important in cancer development and progression.

Taken together, while we are beginning to understand the molecular and cellular consequences of variants in the U5 snRNP proteins and how they relate to human disease and cancer, further research is required to understand the tissue specificity of these disorders, the distinct phenotypes arising from variants in interacting proteins of the same spliceosome complex, and the pleiotropic phenotypes arising from different changes in the same U5 snRNP factor.

## Author Contributions

KW performed the literature review and writing of the manuscript. ME produced Figure 1. WN and RO’K performed the supervision, discussion, and proofreading and editing. All authors contributed to the article and approved the submitted version.

## Conflict of Interest

The authors declare that the research was conducted in the absence of any commercial or financial relationships that could be construed as a potential conflict of interest.

## Footnotes

1 https://sph.uth.edu/retnet/
